# A Cross-Sectional Study of the Prevalence of Metabolic Syndrome among Young Female Emirati Adults

**DOI:** 10.1371/journal.pone.0159378

**Published:** 2016-07-14

**Authors:** Ayesha S. Al Dhaheri, Maysm N. Mohamad, Amjad H. Jarrar, Eric O. Ohuma, Leila Cheikh Ismail, Fatima T. Al Meqbaali, Usama Souka, Syed M. Shah

**Affiliations:** 1 Nutrition and Health Department, College of Food and Agriculture, United Arab Emirates University, Al Ain, UAE; 2 Centre for Statistics in Medicine, Nuffield Department of Orthopaedics, Rheumatology & Musculoskeletal Sciences, University of Oxford, Oxford, United Kingdom; 3 Centre for Tropical Medicine and Global Health, Nuffield Department of Medicine, University of Oxford, Oxford, United Kingdom; 4 Nuffield Department of Obstetrics & Gynaecology and Oxford Maternal & Perinatal Health Institute, Green Templeton College, University of Oxford, Oxford, United Kingdom; 5 Institute of Public Health, College of Medicine and Health Sciences, United Arab Emirates University, Al Ain, UAE; Dasman Diabetes Institute, KUWAIT

## Abstract

**Introduction:**

Metabolic syndrome (MetS) is a growing problem in the United Arab Emirates (UAE). Moreover, the prevalence of overweight and obesity is rapidly increasing in the UAE especially among young females. However, few studies have evaluated the prevalence of MetS among young female adults in the UAE. This study determined the prevalence of MetS in Emirati females aged 17–25 years and its relation to overweight and obesity.

**Methods:**

In total, 555 Emirati female college students were enrolled in a cross-sectional study, conducted during 2013–2014 at United Arab Emirates University in Al Ain, UAE. Anthropometric measurements, blood pressure and biochemical measurements were collected. MetS was defined according to the harmonised International Diabetes Federation criteria.

**Results:**

Of the 555 participants enrolled, 23.1% were overweight and 10.4% were classified as obese. The overall prevalence of MetS was 6.8%. MetS prevalence was highest among obese participants (34.5%), as compared with normal-weight (1.7%) and overweight (10.1%) participants. MetS was significantly associated with overweight (adjusted odds ratio [aOR] = 3.8, 95% confidence interval [CI]; 1.15–12.52) and obesity (aOR = 11.2, 95% CI; 3.1–40.9), as compared with normal-weight. Waist-hip ratio ≥ 0.8 (aOR = 3.04, 95% CI; 1.10–8.44) was significantly associated with MetS, as compared with waist-hip ratio <0.8. The odds of MetS were 22 fold higher in participants with glycated haemoglobin (HbA1c) ≥ 6.5% (aOR = 22.5, 95% CI; 6.37–79.42) compared to HbA1c <6.5%. This difference was 9 fold higher when HbA1c between 5.6%–6.4% was compared to HbA1c <5.6% (aOR = 8.9, 95% CI; 3.4–23.5).

**Conclusion:**

The prevalence of MetS among obese Emirati female students was significantly higher than overweight and normal weight students. The high prevalence of MetS highlights the importance of regular screening and intervention programmes targeting weight reduction.

## Introduction

Non-communicable diseases (NCDs) are the leading cause of deaths worldwide, and diabetes mellitus (DM) is the fourth major cause of NCD deaths [1].

A diagnosis of metabolic syndrome (MetS) is based on the existence of pre-diabetes combined with dyslipidaemia (elevated levels of total or low-density lipoprotein [LDL] cholesterol, or low high-density lipoprotein [HDL] cholesterol levels), elevated blood pressure and obesity [2]. Based on the International Diabetes Federation (IDF) definition for MetS; a study conducted in Emirati adults (>20 years old) by Malik and Razig in 2008 reported the total MetS prevalence was 40.5% and was higher among women (45.9%) than men (32.9%) [3]. The study by Mehairi et al. in 2013 showed an increase in the prevalence of MetS with higher body mass index (BMI) values [4].

The lifestyle of the Emirati population has changed considerably over the past 40 years due to the rapid improvement in socioeconomic status. This transition has led to less physical activity and altered eating habits. These changes, in addition to the adoption of a western lifestyle and diet, have led to the rise in the prevalence of overweight and obesity in the UAE, particularly among females [5]. There is a paucity of data available about the prevalence of MetS and its relation with overweight and obesity among young female adults in the UAE. Moreover, the population structure of UAE is mainly young and has therefore been greatly affected by the rapid socioeconomic changes.

This study aimed to determine the prevalence of MetS in Emirati females aged 17–25 years as this age range has not been studied previously, and its relation to overweight and obesity in Al Ain, UAE.

## Design and Methods

### Study population

A cross-sectional population-based study was conducted during the academic year 2013/ 2014 at United Arab Emirates University (UAEU) in Al Ain, UAE. The university currently enroll around 14,000 students each year.

The study population included students from all nine colleges of the university. Participants were asked to read the information sheet carefully and were given the chance to ask any question related to the study before providing written informed consent to participate. Each participant was assigned a personal identification number to maintain anonymity and data confidentiality. Ethical approval was obtained from the United Arab Emirates University Scientific Research Ethics Committee (Reference number DVCRGS/370/2014).

**Fig 1:**
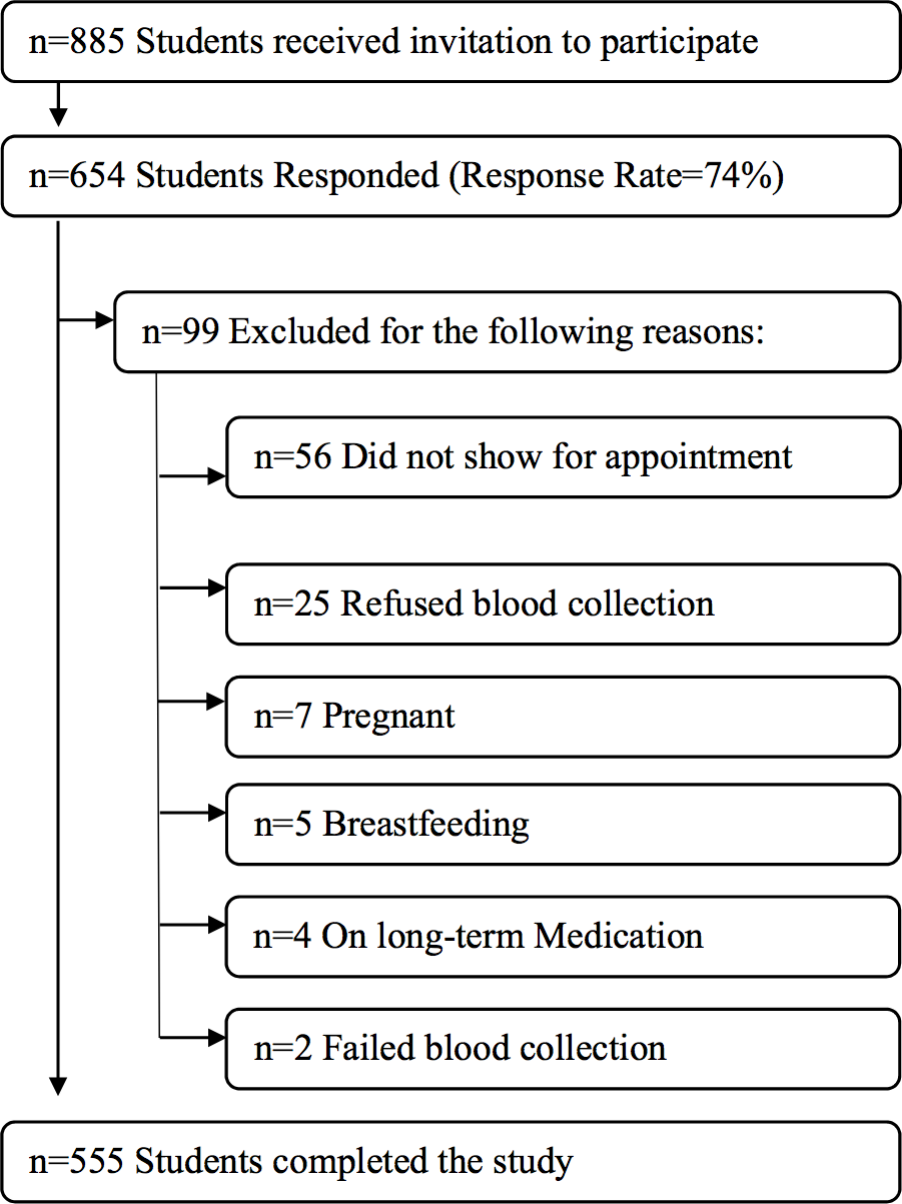
Study participants enrolment process

A stratified random sampling approach was used to select eligible participants [6]. All female students were divided into strata by college (nine strata), and then a random subsample proportional to size that consisted of 10% of the students from each college were selected. All eligible students were then contacted via e-mail to request their participation in the study. Of the total number of females registered in the university during the 2013/2014 academic year (n = 8846), a subsample of 885 students received a request to participate in the study. The enrolment process of the study participants is shown in Fig 1.

### Questionnaire

Each participant completed a self-reported questionnaire on demographic data, supplements and medication use, tobacco use, diet, physical activity, sleeping patterns, perceptions about obesity, personal history of NCDs and family history (first-degree relatives) of NCDs. Participants completed the questionnaire under the supervision of the research team to respond to any clarification needed on any aspects of the questionnaire or the study as a whole.

### Anthropometric measurements and physical examination

Height was measured using a portable stadiometer (Seca Stadiometer, Seca Ltd, Birmingham, UK), in the standing position, without shoes and recorded to the nearest millimetre [7]. Body weight (Kg) and body composition were measured using the Tanita Segmental Body Composition Analyser (Tanita BC-418, Tanita Corp., Tokyo, Japan) [8]. The World Health Organization classification of the BMI (weight / height^2^; (kg/m^2^)) was used to classify underweight, normal-weight, overweight and obesity in the studied population [9]. Waist circumference (WC) was measured in centimetres (cm) using a plastic tape, at the midway between the inferior margin of the ribs and the superior border of the iliac crest or at umbilicus level for obese participants [10]. Hip circumference (HC) was measured at the level of maximum posterior extension of the buttocks [11].Waist-hip ratio (WHR) was calculated by dividing WC by HC. Total body fat was measured from skin-fold thickness at four sites (biceps, triceps, subscapular and suprailiac) using the equation described by Durnin and Womersley in 1974 [12]. Cut-off points for body fat percentage, WHR, and anaemia were based on World Health Organization recommended values [13–15].

The anthropometric measurements were carried out by a trained anthropometrist to reduce inter-observer variations. All measurements were completed during a single 50-minute session (to eliminate missing data), with the participants reporting to the clinic having fasted for 12–14 hours prior to testing, although drinking water was allowed in moderation. Measurements were taken in the morning between 7:00–10:00 a.m. to minimize inter-day fluctuations. Participants were asked not to visit the clinic during their menstrual cycle. Participants were encouraged to rest for 15 minutes before any measures were performed to enable them to relax before performing any of the tests. Each measurement was taken three times and averaged to improve accuracy. All measuring devices were calibrated on a daily basis.

Blood pressure (BP) was measured by a registered nurse using a validated and calibrated digital automated sphygmomanometer (Omron Hem-907, Omron Healthcare, Kyoto, Japan), after the participant had rested for at least 15 minutes [16]. Two consecutive measurements were obtained 5-minutes apart and the average of the two readings recorded [17].

### Laboratory measurements

A registered nurse collected a 5-ml venous blood sample from each participant after 12 hours of fasting via a vacuum system (vacuette 0.64 × 19mm, Greiner Bio-One, Kremsmünster, Austria), into a serum separator tube with clot activator (Vacutest Kima srl, Arzergrande, Italy). Blood samples were centrifuged (2,500 rpm, 15 minutes) and the serum was properly separated, identified and stored at −80°C until the time of analyses.

Samples were thawed on ice for 30 minutes with proper handling during thawing and storage [18]. The total cholesterol, triglyceride (TG), LDL-cholesterol, HDL-cholesterol (HDL-C), high-sensitivity C-reactive protein and fasting blood glucose concentrations in human serum analyses were performed using the Cobas C111 automated biochemical analyser (Roche Diagnostics, Indianapolis, IN, USA) [19]. The HemoCue Hb 201+ portable photometer system (HemoCue AB, Ängelholm, Sweden) was used for the assessment of haemoglobin concentration and the HemoCue HbA1c 501 system was used for assessing glycated haemoglobin (HbA1c) percentage in whole blood. MetS was defined according to the harmonised definition established in 2009 by the IDF and the American Heart Association/the National Heart, Lung, and Blood Institute (AHA/NHLBI) as the presence of any three of the following five factors: elevated WC ( ≥ 80 cm in women); hypertriglyceridaemia (TG ≥ 150 mg/dL or drug treatment for elevated TG); reduced HDL-C (<50 mg/dL in women or drug treatment for reduced HDL-C); elevated BP (systolic BP ≥130 mmHg and/or diastolic BP ≥85 mmHg or use of antihypertensive drugs); and elevated fasting blood glucose ≥100 mg/dL or use of hypoglycaemic medication) [20].

### Sample size calculation

Sample size was calculated using the Minitab software (version 16, Minitab Inc, PA, State College, USA) and was based on an expected prevalence of 4%. At 80% power and 5% significance level, a sample size of 555 would achieve a 1.58% margin of error for the survey of the female student population.

### Statistical analyses

Data analyses were carried out using Stata version 13 (Stata Corp, College Station, TX, USA). Descriptive statistics were computed and summarised; continuous variables were summarised using means and standard deviations (SD) and categorical variables using proportions. The Student’s *t*-test was used for continuous variables to compare mean differences between participants with and without MetS. Univariable and multivariable logistic regression analysis was used to study the association between anthropometric and chemical measures and the presence or absence of MetS as the outcome variable. To account for perfect prediction of MetS by BMI categories and the small sample size across BMI class, we applied the Firth logistic regression to obtain reasonable and robust estimates. All statistical significance was assessed at the 5% significance level.

## Results

Of the 885 students invited to participate, the response rate was 74% (n = 654). The overall prevalence of MetS was 6.8% (95% CI: 5% to 9%). The demographic and clinical characteristics of the study population by MetS status are presented as mean ± SD, in Table 1. The mean age of the study population was 20.4 ± 1.7 years. The average age of participants with MetS was not significantly different from those without MetS (20.9 vs. 20.4 years, *P* = 0.057). However, participants with MetS had a significantly higher weight, height, HC, BMI, body fat percentage, serum LDL (mg/dL) and HbA1c level (*P*< 0.01).

**Table 1:** Demographic and clinical characteristics by metabolic syndrome status.

	With Metabolic Syndrome (N = 38) Mean ± SD	Without Metabolic Syndrome (N = 517) Mean ± SD	*Student’s t-test P*-value
**Age (Year)**	20.9 ± 1.7	20.4 ± 1.7	0.057
**Weight (Kg)**	82.1 ± 17.1	58.9 ± 12.2	< 0.001
**Height (cm)**	161.3 ± 5.2	158.9 ± 5.8	0.013
**Hip Circumference (cm)**	115.42 ± 12.35	98.67 ± 9.71	<0.001
**Body Mass Index (Kg/m**^**2**^**)**	31.5 ± 6.3	23.2 ± 4.6	<0.001
**Body Fat (%)**	40.3 ± 3.9	32.9 ± 5.3	<0.001
**Serum Low Density Lipoprotein (mg/dL)**	102.5 ± 30.9	91.0 ± 24.7	0.006
**Serum Total Cholesterol (mg/dL)**	165.7 ± 37.9	155.6 ± 31.9	0.063
**Glycated Haemoglobin (%)**	6.3 ± 1.1	5.5 ± 0.8	<0.001
**Haemoglobin (g/dL)**	12.00 ± 1.6	11.8 ± 1.5	0.482

**Fig 2:**
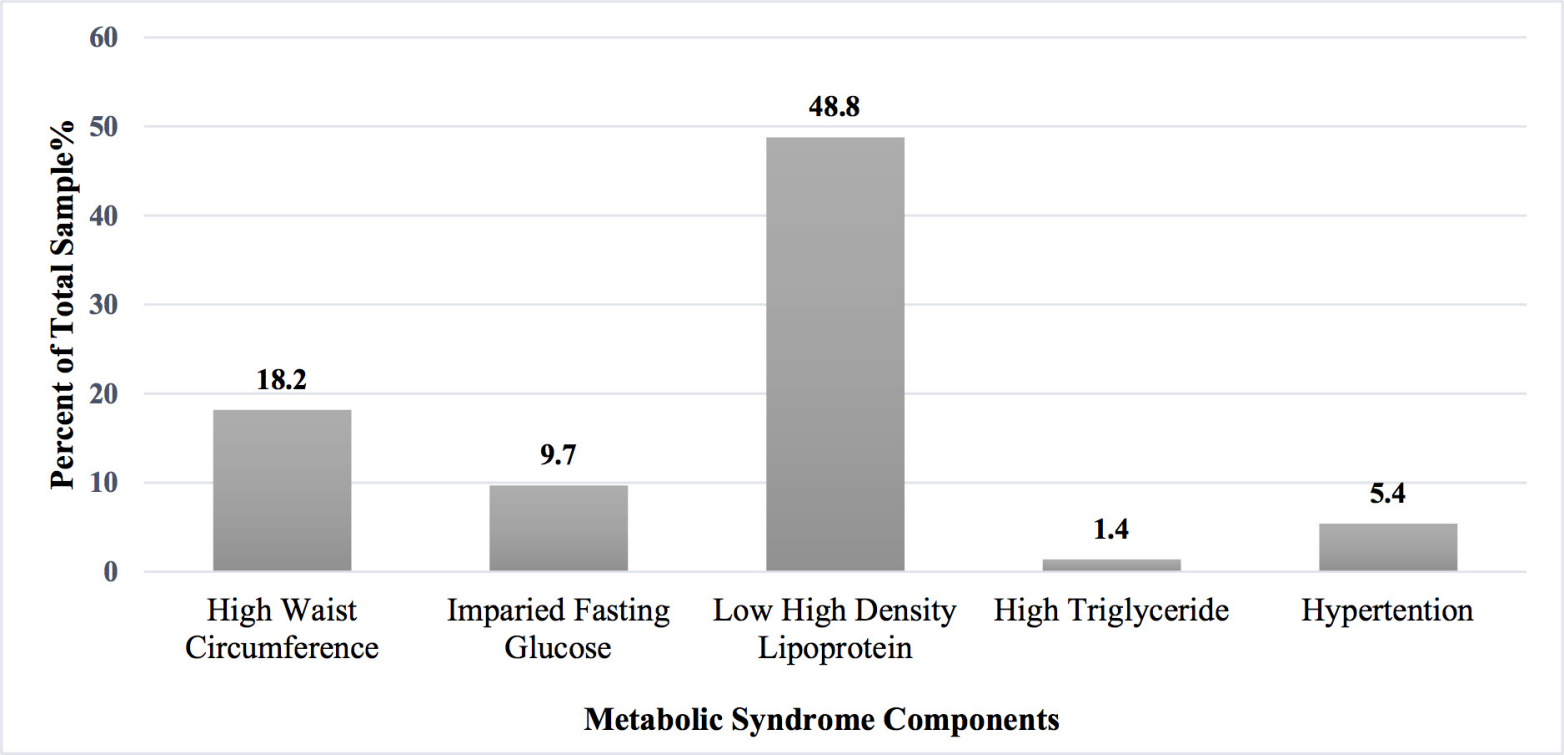
Prevalence of metabolic syndrome components among UAEU young female adults 17–25 years (N = 555), Al Ain, UAE.

No MetS defining components were found in 242 (43.6%) participants. At least one MetS component was found in 213 participants (38.4%); two MetS components were present in 62 participants (11.2%); three MetS components were found in 27 participants (4.9%); four components of MetS were present in 10 participants (1.8%); and all five MetS components were found in only one participant (0.2%). The most frequent component of MetS was reduced HDL-C levels (48.8%), followed by central obesity (18.2%) and impaired fasting glucose (9.7%) (Fig 2).

**Fig 3:**
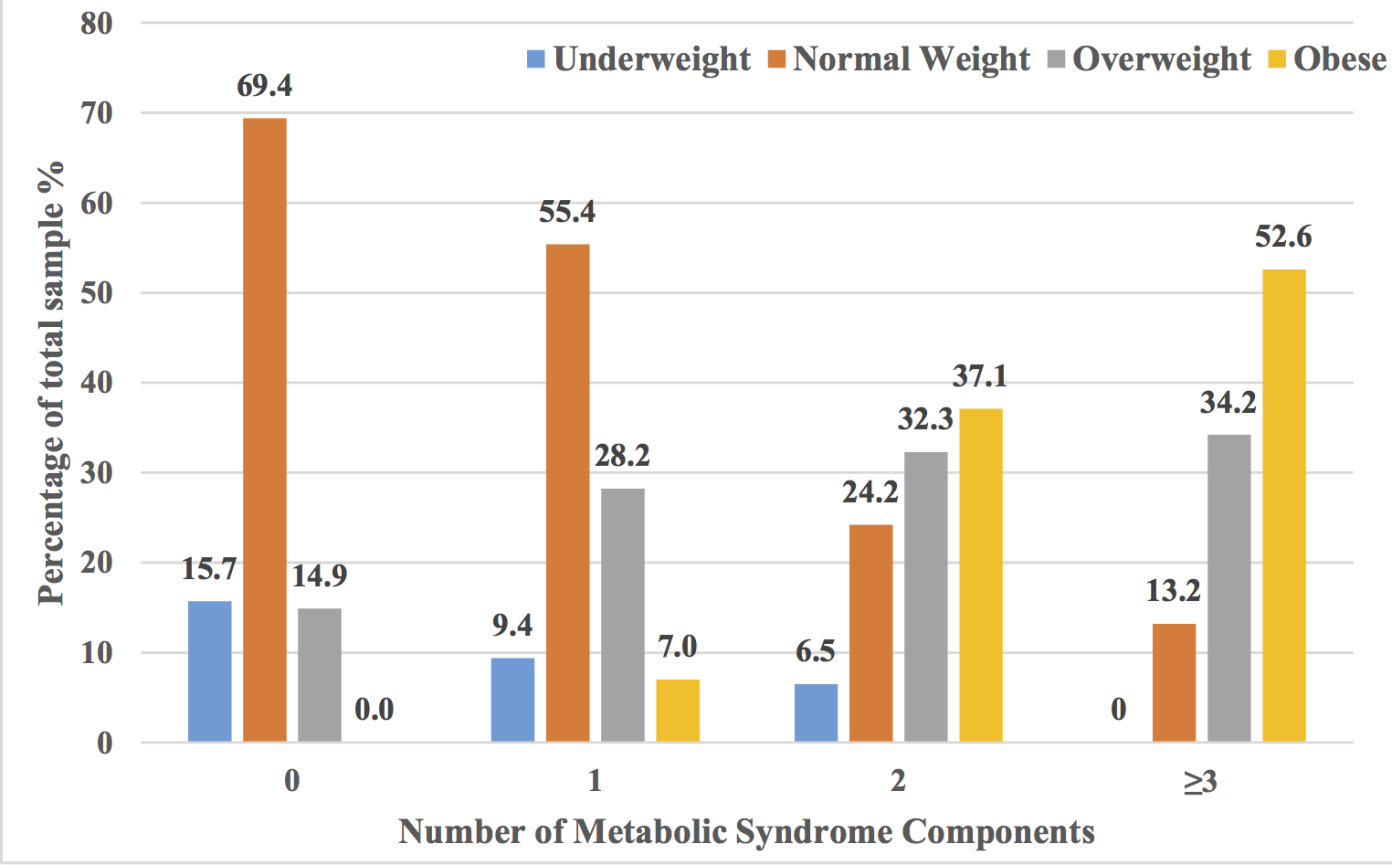
Percentage of participants per number of metabolic syndrome components and BMI category among young female adults aged 17–25 years (n = 555), Al Ain, UAE.

A Chi-square test of association between MetS and BMI categories among young female adults showed a statistically significant association (*P* < 0.001), and was particularly high among obese participants (34.5%) compared to 10.1% overweight, and 1.7% normal-weight. None of the five MetS components were observed in 69% of the normal-weight participants whereas all obese participants had at least one MetS component. Obese participants were more likely to have three or more MetS components (52.6%) than overweight (34.2%) and normal-weight (13.2%) participants. Furthermore, none of the underweight participants had three or more MetS components (Fig 3).

Table 2 shows the univariable and multivariable logistic regression results for the odds of MetS by potential risk factors. In the univariable analyses, older participants (23–25 years) were three times more likely to have MetS (odds ratio [OR]: 2.96; 95% CI: 1.03 to 8.52) than younger participants (17–19 years) but this effect was not significant in the multivariable analyses (odds ratio [OR]: 1.14; 95% CI: 0.30 to 4.30). Participants who reported having a family history of diabetes or hypertension (n = 305) had a 2.8 times elevated risk of MetS (OR: 2.81; 95% CI: 1.31 to 6.06) compared with participants without a family history of diabetes or hypertension in the univariable analyses but not in the adjusted analyses (OR: 1.85; 95% CI: 0.73 to 4.65). Participants who were overweight or obese were, respectively, 6.4 (95% CI; 2.3 to 17.5) and 29 (95% C1; 29.2 to 79.3) times more likely to have MetS than those of normal-weight in the univariable analysis. These findings remained significant even after adjusting for other potential confounders (i.e. OR = 3.8; 95% CI: 1.15–12.52 for overweight; and OR = 11.2; 95% CI: 3.06–40.86 for obese participants). Participants with percentage body fat ≥35% (n = 227) showed a significantly higher risk for the development of MetS in the univariable analyses (OR: 14.27; 95% CI: 4.99 to 40.83; *P*<0.001), but the difference was not significant after adjusting for other factors (OR = 3.12; 95% CI: 0.91–10.68). A WHR of more than 0.8 was significantly associated with at least three times increased risk of MetS (*P*<0.001) in the adjusted analyses when compared with those with a WHR <0.8 (aOR = 3.04; 95% CI: 1.10–8.44). Elevated HbA1c (≥6.5%) showed a high significant association with the presence of MetS (OR: 14.15; 95% CI: 4.78 to 41.86; *P*<0.001) in univariable analyses and remained significant in the adjusted analyses (adjusted OR [aOR]: 22.49, 95% CI: 6.37 to 79.42; *P*<0.001). Total cholesterol ≥200 mg/dL and LDL ≥130 mg/dL conferred a greater likelihood for MetS: OR: 3.34 (95% CI: 1.48 to 7.49) and OR: 1.86 (95% CI: 0.96 to 3.63), respectively, in the univariable analyses but not in the adjusted analyses.

**Table 2:** Risk factors for MetS among young female adults aged 17–25 years (n = 555), Al Ain, UAE.

Characteristics	Sample	n (%)	With MetS			
			Crude Odds Ratio (95%CI)	P-value	Adjusted Odds Ratio (95%CI)	*P*-value
**Age (Year)**						
17–19	194	8 (4.1)	Reference		Reference	
20–22	299	23 (7.7)	1.94 (0.85, 4.42)	0.116	1.89 (0.72–4.94)	0.19
23–25	62	7 (11.3)	2.96 (1.03, 8.52)	0.044	1.14 (0.30–4.30)	0.85
**Family history of diabetes or hypertension (%)**						
No	250	9 (3.6)	Reference		Reference	
Yes	305	29 (9.5)	2.81 (1.31, 6.06)	0.008	1.85 (0.73–4.65)	0.19
**Body Mass Index (Kg/m**^**2**^**)**						
Underweight (<18.5)	62	0 (0.0)	0.44 (0.02, 8.03)	0.58	0.85 (0.04–17.26)	0.92
Normal-weight (18.5–<25)	306	5 (1.6)	Reference		Reference	
Overweight (25–29.9)	129	13 (10.1)	6.35 (2.30, 17.51)	<0.001	3.80 (1.15–12.52)	0.028
Obese (≥30.0)	58	20 (34.5)	29.19 (10.75, 79.28)	<0.001	11.19 (3.06–40.86)	<0.001
**Body Fat (%)**						
<35%	328	4 (1.2)	Reference		Reference	
≥35%	227	34 (14.9)	14.27 (4.99, 40.83)	<0.001	3.12 (0.91–10.68)	0.07
**Waist-Hip Ratio**						
<0.8	509	28 (5.5)	Reference		Reference	
≥0.8	46	10 (21.7)	4.77 (2.15, 10.59)	<0.001	3.04 (1.10–8.44)	0.033
**Anaemia**						
No	271	22 (8.1)	Reference		Reference	
Yes	284	16 (5.6)	0.67 (0.35, 1.32)	0.249	1.04 (0.46–2.35)	0.92
**Total Cholesterol (mg/dL)**						
< 200	502	29 (5.8)	Reference		Reference	
≥200	53	9 (16.9)	3.34 (1.48, 7.49)	0.004	1.71 (0.53–5.55)	0.37
**Low Density Lipoprotein (mg/dL)**						
<130	380	21 (5.5)	Reference		Reference	
≥ 130	173	17 (9.8)	1.86 (0.96, 3.63)	0.067	0.92 (0.37–2.32)	0.86
**Glycated Haemoglobin (%)**						
< 5.6	374	6 (1.6)	Reference		Reference	
5.6–6.4	133	23 (17.3)	12.82 (5.09, 32.29)	<0.001	8.92 (3.39–23.48)	<0.001
≥ 6.5	48	9 (18.8)	14.15 (4.78, 41.86)	<0.001	22.49 (6.37–79.42)	<0.001

A subgroup analysis was conducted for the study population excluding all females with HbA1c > = 6.5 resulting in a total sample size of 507 participants. Results of the subgroup analysis remain largely unchanged based on the magnitude of the effect sizes, direction of significance and overall conclusions. However, in the multivariate analysis, only waist hip-ratio was no longer significant in the subgroup analysis (OR = 2.12; 95% CI: 0.65–6.87; *P* = 0.211)

## Discussion

In the United Arab Emirates (UAE) rapid socioeconomic growth has resulted in profound lifestyle changes including sedentary behaviours, westernized diets and increased energy intake [5]. The prevalence of MetS among Emirati females has been reported to be higher than that for Emirati males in the adult population (32.9% among men, 45.9% among women) [3]. This research highlights the importance of investigating MetS among young female adults, to facilitate understanding of the prevalence and risk factors of MetS. There is paucity of data on the prevalence of MetS among Emirati females aged 17–25 years. The current study reveals a MetS prevalence of 6.8% among young female Emirati adults aged 17–25 years, and 34.5% among young obese female Emirati adults.

The results of the current study are in line with findings among college students (18–26 years) in Saudi Arabia [21], where the overall MetS prevalence was 7.8%, and 26.4% in obese students. In Kuwait, the prevalence of MetS was even higher among female adolescents (10–19 years) at 9.1% and 14.8% according to ATP III and IDF criteria, respectively [22]. These numbers are certainly close to those described in the UAE, which is not surprising, considering the relatively similar rapid increase in obesity and diabetes rates throughout the Gulf region [23, 24]. These trends are likely to be a result of the sedentary and westernised lifestyle [25–28], and could also be partially explained by the “thrifty genes” hypothesis [29], which suggests that the genotype of mankind existed as hunter-gatherers can efficiently store food in the adipose tissue during periods of food abundance, to compensate for periods of food shortage.

Worldwide, the prevalence of MetS among young female adults in the USA (18–21 years) was 4.7% [30], in Brazilian college students it was 1.7% [31], in Chinese female adolescents (14–16 years) it was 2.5% [32], in Spanish female adolescents (10–15 years) it was 3.85% [33], in Tunisian female adolescents (10–19 years) it was 2.4% [34], and 11.7% among Indian female adolescents (10–19 years) [35]. Clearly, the prevalence of MetS could differ between countries depending on the MetS defining criteria used, study method and target population. We have used the IDF and AHA/NHLBI joint statement, as it was an international attempt to harmonise the definition of MetS; central obesity is not an obligatory component of this definition and it is ethnic specific.

The most frequent component of MetS in this study was reduced HDL-C levels, which was also reported in female Emirati adolescents [4], and female Kuwaiti adolescents [22]. Reduced HDL-C accompanied by elevated triglyceride levels indicates dyslipidaemia, which is highly prevalent among the UAE population [36]. Insufficient physical activity and poor dietary habits are associated with low HDL-C levels [37–39]. Elmagd et. al. [40] reported low physical activity (defined as less than 150 minutes/week) in 60% of Emirati college students. The consumption of high caloric diet was also reported in 33.5% of female Emirati adolescents [5]. These findings could explain the high prevalence of low-HDL-C levels observed in our study. Regular checks and screening in this age group could be helpful in identifying participants at an increased risk of developing MetS.

The current study found strong correlations between BMI, body fat, HbA1c and the prevalence of MetS in Emirati female students. The relationship between overweight and obesity and MetS has been supported by many other studies [41–43]. The association between HbA1c and the prevalence of MetS has not been previously reported; nevertheless, insulin resistance is a major underlying mechanism accountable for the prevalence of the MetS [44]. Interestingly, the prevalence of diabetes (8.5%) was also high in the study population. Future studies need to explore this finding more closely.

The strengths of this study include a trained researcher who obtained all measurements in the study and each measurement was repeated three times and the average used in the analyses. Anthropometric measures and blood withdrawal were conducted during one 50-minute morning session after assurance of a 12-hour overnight fast. Furthermore, to the best of our knowledge, no other studies exploring MetS prevalence in college students have been conducted in the UAE. UAEU is the main university in the UAE and it enrols students from all seven emirates. However, restricting the study to college students makes it not representative of all the Emirati females in this age group. Moreover, the cross-sectional design is another limitation of this study, as causal inference cannot be drawn. Participants were voluntarily enrolled in the study, which could have caused selection bias (overweight and obese individuals might avoid anthropometric measurements). In addition, studying female students only does not allow for examination of gender differences or generalisability of results to all young adults. Therefore, future prospective studies are needed to confirm the prevalence of MetS and its relation to overweight and obesity in Emirati young adults. Additionally, it was challenging to clearly define the “young adult” age group. Some studies reported the MetS prevalence in adolescents and included ages 12–18 years [4] or 10–19 years old [22]. Other studies defined young adults as 18–24 years [30], 17–37 years [45] or college students aged 17–25 years [46]. Having one international definition for the “young adult” age group would be helpful for future data comparisons.

## Conclusions

In summary, we have shown that the prevalence of MetS is high among UAEU female young adults aged 17–25 years (6.8%). Identification and possible intervention programmes maybe useful for this age group in order to improve their future health. In addition, reduced HDL-C levels followed by central obesity were the most frequent components of MetS. BMI, body fat percentage and HbA1c were significantly associated with MetS.
